# Integration of single-cell RNA-seq and bulk RNA-seq to construct liver hepatocellular carcinoma stem cell signatures to explore their impact on patient prognosis and treatment

**DOI:** 10.1371/journal.pone.0298004

**Published:** 2024-04-18

**Authors:** Lixia Liu, Meng Zhang, Naipeng Cui, Wenwen Liu, Guixin Di, Yanan Wang, Xin Xi, Hao Li, Zhou Shen, Miaomiao Gu, Zichao Wang, Shan Jiang, Bin Liu

**Affiliations:** 1 Department of Ultrasound and Hebei Key Laboratory of Precise Imaging of Inflammation Related Tumors, Affiliated Hospital of Hebei University, Baoding, 071052, China; 2 Department of Hepatobiliary Surgery, Affiliated Hospital of Hebei University, Baoding, 071052, China; 3 Department of Breast Surgery, Affiliated Hospital of Hebei University, Baoding, 071052, China; 4 Department of Ultrasound, Affiliated Hospital of Hebei University, Baoding, 071052, China; 5 Department of Pathology, Affiliated Hospital of Hebei University, Baoding, 071052, China; 6 Central Laboratory, Hebei Key Laboratory of Cancer Radiotherapy and Chemotherapy, Affiliated Hospital of Hebei University, Baoding, 071052, China; Xiangya Hospital Central South University, CHINA

## Abstract

**Background:**

Liver hepatocellular carcinoma (LIHC) is a prevalent form of primary liver cancer. Research has demonstrated the contribution of tumor stem cells in facilitating tumor recurrence, metastasis, and treatment resistance. Despite this, there remains a lack of established cancer stem cells (CSCs)-associated genes signatures for effectively predicting the prognosis and guiding the treatment strategies for patients diagnosed with LIHC.

**Methods:**

The single-cell RNA sequencing (scRNA-seq) and bulk RNA transcriptome data were obtained based on public datasets and computerized firstly using CytoTRACE package and One Class Linear Regression (OCLR) algorithm to evaluate stemness level, respectively. Then, we explored the association of stemness indicators (CytoTRACE score and stemness index, mRNAsi) with survival outcomes and clinical characteristics by combining clinical information and survival analyses. Subsequently, weighted co-expression network analysis (WGCNA) and Cox were applied to assess mRNAsi-related genes in bulk LIHC data and construct a prognostic model for LIHC patients. Single-sample gene-set enrichment analysis (ssGSEA), Cell-type Identification By Estimating Relative Subsets Of RNA Transcripts (CIBERSORT) and Tumor Immune Estimation Resource (TIMER) analysis were employed for immune infiltration assessment. Finally, the potential immunotherapeutic response was predicted by the Tumor Immune Dysfunction and Exclusion (TIDE), and the tumor mutation burden (TMB). Additionally, pRRophetic package was applied to evaluate the sensitivity of high and low-risk groups to common chemotherapeutic drugs.

**Results:**

A total of four genes (including *STIP1*, *H2AFZ*, *BRIX1*, and *TUBB*) associated with stemness score (CytoTRACE score and mRNAsi) were identified and constructed a risk model that could predict prognosis in LIHC patients. It was observed that high stemness cells occurred predominantly in the late stages of LIHC and that poor overall survival in LIHC patients was also associated with high mRNAsi scores. In addition, pathway analysis confirmed the biological uniqueness of the two risk groups. Personalized treatment predictions suggest that patients with a low risk benefited more from immunotherapy, while those with a high risk group may be conducive to chemotherapeutic drugs.

**Conclusion:**

The current study developed a novel prognostic risk signature with genes related to CSCs, which provides novel ideas for the diagnosis, prognosis and treatment of LIHC.

## Introduction

Liver cancer is the third most frequent cause of cancer-related death [1,2]. Treatment options for liver hepatocellular carcinoma (LIHC) are rapidly increasing and include surgery, chemotherapy, immunotherapy, and hepatic artery embolization [3,4]. However, the treatment of LIHC remains challenging, as LIHC heterogeneity has been demonstrated to be a huge obstacle to LIHC treatment [5]. Hence, there is an urgent need for suitable prognostic characterization based on single cell level to help provide potential therapeutic targets for LIHC.

Cancer stem cells (CSCs) are very few immature malignant cells [6]. CSCs are thought to have the potential for self-renewal and plasticity in a variety of cancers, including LIHC, and play a key role in tumor recurrence, metastasis, and treatment resistance [7–9]. In detail, CSCs can experience epithelial-to-mesenchymal transition (EMT), invasion, circulation in the bloodstream and distant exudation, forming metastatic lesions [9]. In addition, CSCs also promote DNA repair and cut down apoptosis compared to bulk tumor cells [10]. In another way, Zheng et al. showed that CSCs in LIHC are functionally, phenotypically, and transcriptionally heterogeneous at the single-cell level, and that genes in different CSC subpopulations are independently associated with prognosis in LIHC [11]. Therefore, further exploration of CSCs in LIHC is necessary. CytoTRACE is a new algorithm based on single-cell RNA sequencing (scRNA-seq) data to predict the differentiation status of malignant cell populations based on CytoTRACE score, an indicator for cell stemness level [12]. Bian et al. demonstrated high possibility of immune evasion in high stem malignant tumor cells of intrahepatic cholangiocarcinoma applying scRNA-seq data with CytoTRACE computerized method [13]. The characteristics of cancer cells at the genome, epigenome, proteome, and transcriptome levels have been proposed as closely related to the loss of differentiated phenotype and the acquisition of stem cell properties in cancer cells [14]. The novel quantification called the tumor progression index assesses the advancement of tumors by utilizing statistical methods to examine extensive data, while also comparing the analytical profiles of embryonic stem cells and tumor cells. This analysis enables the identification of patterns that may be utilized in predictive models. Malta and his colleagues showed that mRNA expression-based stemness index (mRNAsi) scores are closely linked to biological processes in CSCs [14]. Currently, studies have reported the use of mRNAsi scores to identify new biomarkers of CSCs and predicted the prognosis of LIHC patients with One Class Linear Regression (OCLR) algorithm, but we still lack a comprehensive analysis on the pathogenesis of CSCs in LIHC.

In this study, we performed stemness scoring by integrating scRNA-seq and bulk RNA-seq data from LIHC and using CytoTRACE and OCLR algorithms accordingly. Subsequently, weighted co-expression network analysis (WGCNA) and Pearson analysis were utilized to assess mRNAsi-related genes in bulk LIHC data. In combination with CytoTRACE score, a new and well-predicted prognostic model was constructed. Additionally, we evaluated the response to immunotherapy and sensitivity of patients with different LIHC risk groups to common chemotherapy drugs. The current study may provide a novel insight into the role and value of CSCs-related genes in LIHC progression and prognosis.

## Methods

### Data acquisition

The scRNA-seq data from GSE149614 [15] in the Gene Expression Omnibus (GEO) database were obtained. A total of 21 samples from 10 HCC patients were obtained, including 8 normal, 10 tumor, 2 portal vein tumor thrombosis (PVTT) and 1 lymph.

Bulk RNA-seq data were obtained from the Cancer Genome Atlas (TCGA) database [16] and the HCCDB18 database (http://lifeome.net/database/hccdb/home.html). RNA expression profiles, somatic mutation data, and clinical follow-up information for LIHC were obtained from the TCGA-LIHC dataset, and expression data were formatted as log2 (FPKM+1). A total of 365 cases was obtained from the TCGA-LIHC dataset, and 202 cases of LIHC patients were obtained from the HCCDB18 database.

### scRNA-seq data preprocessing and identification of cellular subpopulations

The "Seurat" package [17] performs quality control on the LIHC scRNA-seq data and calculates the percentage of mitochondrial genes using the "PercentageFeatureSet" function. We only retained genes that ranged from 200 to 8000 and expressed in at least 3 cells. At the same time, the content of mitochondrial genes were less than 10% of the cells in order to ensure the high quality of the scRNA-seq data. After treatment, a total of 67,101 cells were obtained for further study. Subsequently, 21 scRNA-seq samples were normalized and hypervariable genes were distinguished according to the "FindVariableFeatures" function. The "ScaleData" function was utilized to scale the genes. In addition, the data were subjected to principal component analysis (PCA) with dim = 20, followed by removing batch effects with harmony package [18]. RunUMAP function was subsequently adopted for further dimensionality reduction (dims = 1:20). Then, all cells were clustered using the "FindNeighbors" and "FindCluster"functions with a resolution of 1. Finally, cell types were annotated with markers from CellMarker database and references.

### Calculation of cell stemness score

The stem cell properties of each cell in the scRNA-seq data were assessed by using the CytoTRACE package with a CytoTRACE score from 0–1 [12]. Higher CytoTRACE scores indicate better cell stemness and less differentiation. Thus, in this study, we regarded a CytoTRACE score close to 0 as low stemness cells and a score close to 1 as high stemness cells.

Similarity between stem cells and tumor cells in bulk RNA-seq data were reflected by the mRNAs index (mRNAsi) based on the expression data. The mRNAsi ranges from 0 to 1, with a value closer to 1 indicating less differentiated cells and stronger properties of stem cells. Based on the One-Class Logistic Regression (OCLR) algorithm, we used the stemness model of the Progenitor Cell Biology Consortium (PCBC, https://progenitorcells.org/) to calculate the mRNAsi of cells from the TCGA-LIHC and HCCDB18 datasets [14,19]. In addition, according to the median, LIHC patients were grouped into mRNAsi high and low groups. The pheatmap package and the ggpubr package [20] were used to plot differences in mRNAsi for prognosis and different clinical features, including clinical grade and stage.

### Identification of mRNAsi-related genes

The "WGCNA" package [21] was employed to establish a gene co-expression network weighted by TCGA-LIHC cohort. WGCNA provides the capability to pinpoint sets of genes exhibiting high covariance and can help discover potential genes that serve as biomarkers and therapeutic targets. This research specifically aimed at identifying gene modules linked to mRNAsi scores in LIHC by utilizing WGCNA, as well as uncovering genes with a strong correlation to mRNAsi scores for further analysis.

### Enrichment analysis of mRNAsi-correlated genes

In order to investigate the possible roles of genes associated with mRNAsi, an analysis on functional enrichment was conducted for this dataset. The "ClusterProfiler" package [22] in R was utilized to analyze the Gene Ontology (GO, http://www. geneontology.org/) [14] and Kyoto Encyclopedia of Genes and Genomes (KEGG, https://www.kegg.jp/kegg/kegg1.html) functions [23] of the modular genes that are most closely linked to mRNAsi. The obtained results were then filtered based on a significance threshold of p < 0.05.

### Construction and verification of prognostic models

Initially, we analyzed the overlap between the portion of the CytoTRACE score correlation exceeding 0.4 and the gene module portion linked to mRNAsi for further examination. Then, univariate Cox regression analyses on the pivotal genes showing prognostic relevance was conducted using the "glmnet" package [24] for LASSO regression analysis. Multivariate Cox regression analysis was further used to identify and select genes that could serve as independent prognostic markers. Subsequently, prognostic models were developed based on these genes. Moreover, to validate the significance of these prognostic genes, various analytical tools such as Kaplan-Meier (KM) curves, disease-specific survival (DSS) curves, receiver operating characteristic (ROC) curves, progression-free interval (PFI) curves, and disease-free interval (DFI) curves were generated using TCGA-LIHC and HCCDB18 data. Finally, a nomogram was constructed using the "rms" package in R [25] to facilitate personalized treatment for LIHC patients.

#### Immune infiltration analysis

Single-sample gene-set enrichment analysis (ssGSEA) was performed to explore the immune status of patients in different risk groups based on the genes expressions of 28 immune cells in previous studies [26,27]. For supplement, the proportions of 22 immune cells by Cell-type Identification By Estimating Relative Subsets Of RNA Transcripts (CIBERSORT) algorithm [28] and the abundance of six common immunocytes was estimated by utilizing the Tumor Immune Estimation Resource (TIMER) 2.0 (https://cistrome.shinyapps.io/timer/) online tool [29].

### Immunotherapeutic response and drug sensitivity analysis

The TIDE algorithm (http://tide.dfci.harvard.edu/query/) was used to evaluate the potential clinical effects of immunotherapy on different risk groups of patients [30]. Based on somatic mutation data in TCGA-LIHCC to assess the correlation of risk scores with tumor mutation burden (TMB). Finally, the sensitivity of different risk groups to common chemotherapeutic drugs was assessed in the "pRRophetic" package in R [31]. Immunomodulator-related genes obtained from previous studies [32] were used to assess the expression level of these genes in different LIHC risk groups.

### Statistical analysis

The data analyses and result generation were performed using R (version 3.6.0). To compare continuous variables between two groups, the Wilcoxon rank-sum test was employed. Additionally, the Chi-square test was conducted to analyze the correlation among classified variables. Survival differences were evaluated utilizing the log-rank test. The Spearman algorithm was used to evaluate all aforementioned correlations. A significance level of p < 0.05 was considered as the statistical threshold. The Sangerbox platform (http://sangerbox.com/home.html) helped process the data in this study.

## Results

### Transcriptome heterogeneity in LIHC stem cells

**Fig 1:**
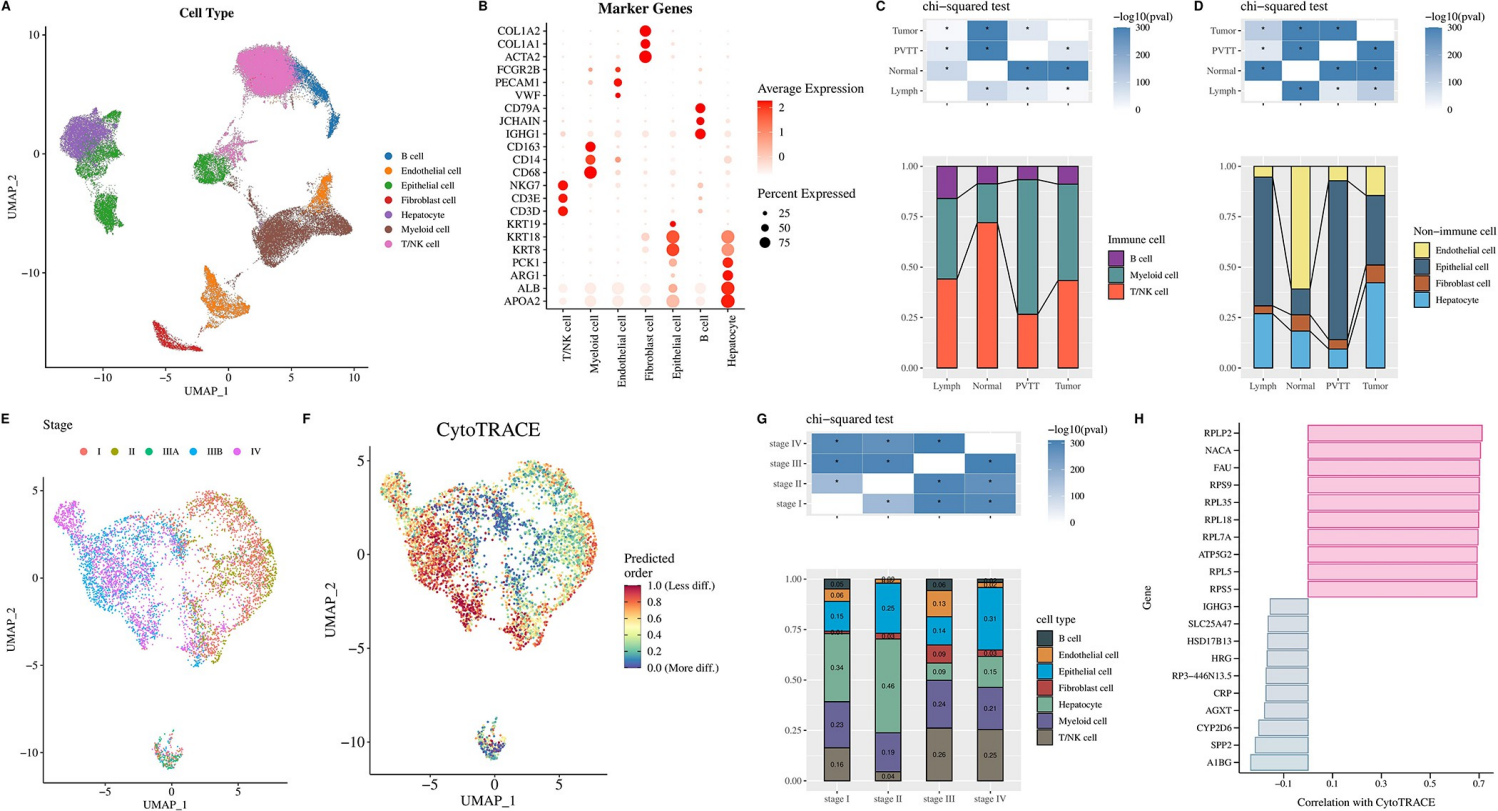
Profiling of cells in LIHC at the scRNA transcript level and cell stemness analysis. Cells were clustered using optimal resolution 1. (A) Seven cell types in LIHC were annotated as the primary markers of the cluster; (B) Expression of marker genes in different cell types; Proportion of immune cells (C) and non-immune cells (D) in scRNA-seq data from LIHC; (E) UMAP plot of the distribution of epithelial cells of primary tumors at different stages; (F) CytoTRACE analysis in primary tumor epithelial cells; (G) Percentage of each cell type in different stages; (H) Top 10 Genes related to CytoTRACE score. *p < 0.05.

We used the scRNA-seq dataset (GSE149614) to select 21 samples from LIHC patients, including 10 tumor samples, 8 normal samples, 2 PVTT samples and 1 lymph sample. Stringent cell quality controls were adopted and 67,101 cells were obtained for subsequent analysis. These 67101 cells were standardized and batch effects were removed, dimensionality was reduced using the "RunUMAP" function, and 25 cell clusters were ultimately identified. Subsequently, we annotated these 25 cell clusters based on cell marker and previous marker genes reported in the literature [15]. As shown in Fig 1A, we obtained a total of 7 cell types, including immune cells (e.g., B cells, myeloid cells, and T/NK cells) and non-immune cells (e.g., endothelial cells, epithelial cells, hepatocytes, and fibroblast cells). In addition, we identified marker genes for each of the seven cell types (Fig 1B). Compared to normal cells, we found that T/NK cells were significantly depleted and myeloid cells were significantly enriched in tumor cells (Fig 1C). In particular, tumor cells in LIHC had a significantly higher proportion of epithelial cells than normal cells (Fig 1D).

In hepatocellular carcinoma tissues, epithelial cells are thought to play a key role in the proliferation of tumor tissue. To eliminate the effect of cells in non-diseased and metastatic tissue samples in the scRNA-seq data of LIHC, we calculated CytoTRACE scores for epithelial cells in LIHC using only the CytoTRACE algorithm. By comparing the differences in CytoTRACE scores of primary tumor epithelial cells between different pathological stages, we observed that highly stemmed tumor cells were predominantly enriched in the late stage (Stage III and IV) (Fig 1E and 1F). As we expected, epithelial cells showed a higher proportion in stage IV than the other six cell types (Fig 1G). In addition, we identified the top10 genes with the highest correlation out of CytoTRACE scores, including RPLP2, NACA, FAU, RPS9, RPL35, RPL18, RPL7A, ATP5G2, RPL5, RPS5 (Fig 1H).

### Analysis of mRNAsi in LIHC in relation to clinical characteristics

**Fig 2:**
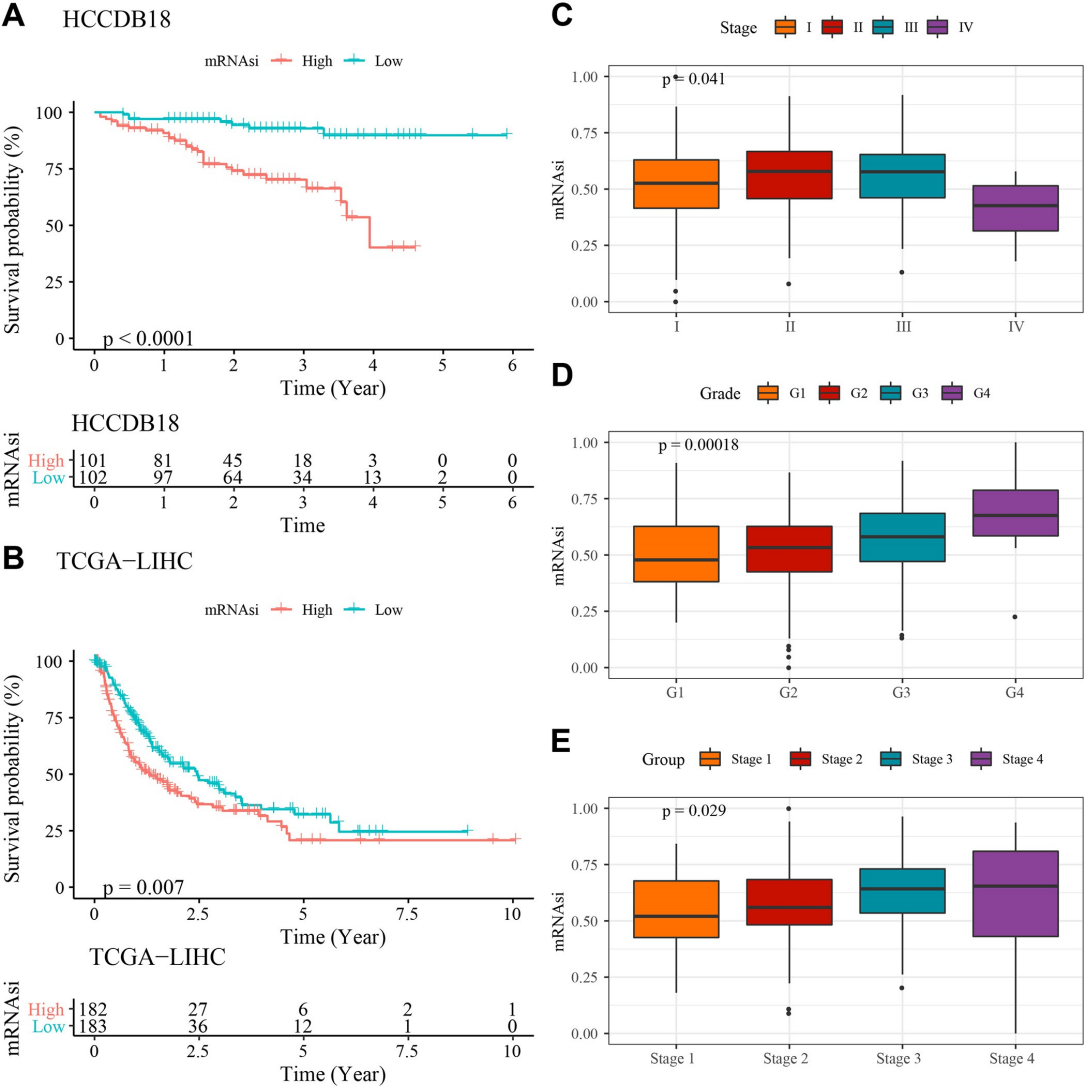
Association of mRNAsi with OS and clinical characteristics of LIHC patients. (A-B) Relationship between mRNAsi and OS in the HCCDB18 (A) TCGA (B) datasets; Association of mRNAsi with tumor stage (C) and grade (D) in the TCGA dataset; (E) Relationship between mRNAsi and tumor stage in the HCCDB8 dataset.

mRNAsi is an indicator that can estimate the number of CSCs by evaluating the similarity and heterogeneity between stem cells and malignant cells. To explore the role of mRNAsi in LIHC, we investigated the effects of mRNAsi on survival of LIHC patients and on LIHC-related clinical characteristics, including Stage and grade, respectively. Based on the KM curves, both in the TCGA-LIHC dataset and the HCCDB18 dataset, we found that elevated mRNAsi expression levels were significantly associated with poor overall survival (OS) in LIHC patients (Fig 2A and 2B). In addition, mRNAsi scores increased with stage (Fig 2E). However, we found that in TCGA-LIHC, the mRNAsi score was lower at Stage IV (Fig 2C). This is most likely because the sample size provied by TCGA database was too small with only 4 samples in IV stage. Fig 2D showed significant differences in mRNAsi among different grades of LIHC, and as the grade increased, the mRNAsi score also increased. This implies that the expression of mRNAsi in LIHC was positively associated with tumor grade.

### Identification of mRNAsi related genes in bulk RNA-seq data using WGCNA

**Fig 3:**
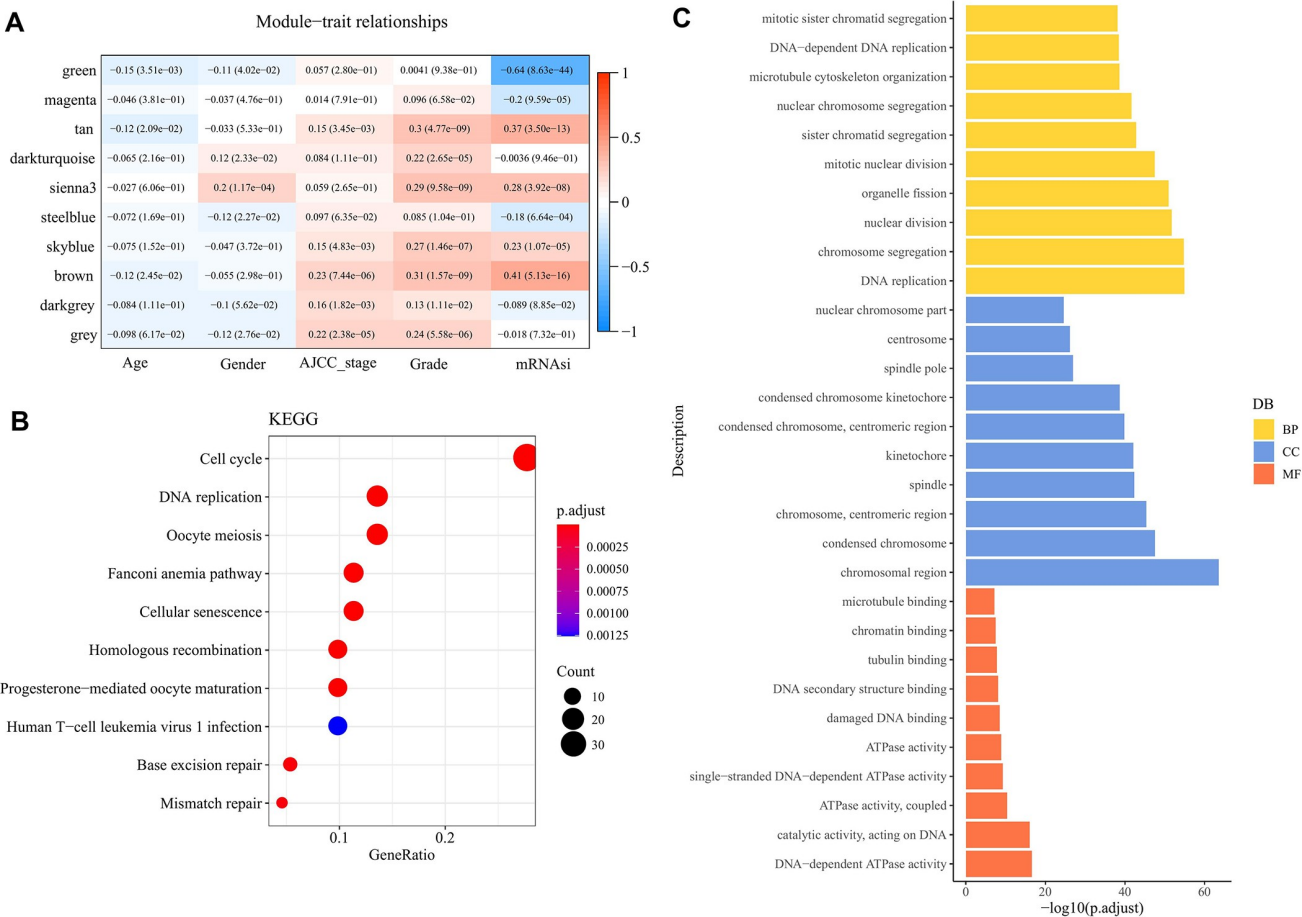
Identification of mRNAsi related genes in LIHC and their enrichment analysis. (A) WGCNA identifies module genes associated with mRNAsi; (B) Top 10 terms for KEGG enrichment analysis of mRNAsi related genes; (C) GO enrichment analysis of mRNAsi related genes.

To identify key genes associated with mRNAsi, we used WGCNA to divide the 13,906 genes in the TCGA-LIHC dataset into 10 gene co-expression module. The expression information of these 10 gene co-expression module is shown in S1 Fig. It could be found that the brown module was significantly positively related to mRNAsi with 349 genes such as TOP2A, MKI67,E2F1, BUB1B etc, which are related to cell cycle (Fig 3A, S2 Fig and S1 Table). Therefore, we defined the genes in the brown module as mRNAsi related genes and further enriched them for analysis.

Results of KEGG enrichment analysis showing the top 10 clusters and their representative enrichment terms. Enriched terms across these mRNAsi related genes were identified for pathways involved in cell cycle, DNA replication, homologous recombination, and cellular senescence (Fig 3B). Additionally, chromosomal regions, chromosome segregation, and damaged DNA binding were significantly regulated by mRNAsi related genes in LIHC, as shown by the results from GO enrichment analysis (Fig 3C).

### Construction of a prognostic model related to CSCs in LIHC

**Fig 4:**
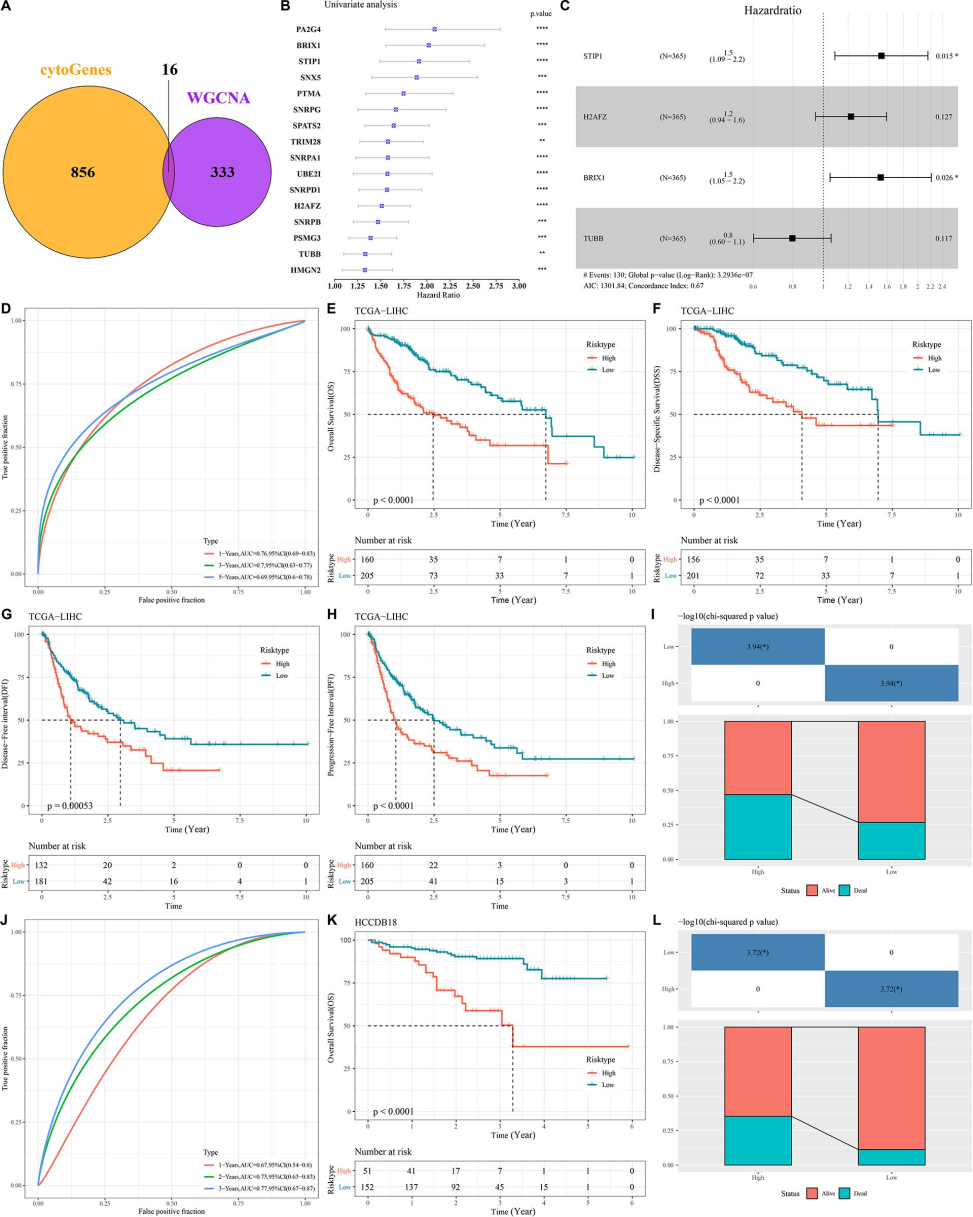
Construction of a prognostic gene risk model associated with CSCs and its survival analysis. (A) Venn plot of CytoTRACE and mRNAsi prognosis-related genes; (B) Univariate analysis of 16 prognostic genes associated with CSCs; (C) Multivariate analysis of 4 prognostically critical genes in LIHC; (D) ROC Curves predict prognosis in LIHC patients at 1,3, and 5 years; (E-H) KM (E), DSS (F), DFI (G), and PFI (H) survival curves based on the TCGA-LIHC cohort; (I) Differences in survival status of patients in different risk groups in the TCGA-LIHC cohort; (J-K) ROC curves (J) and KM curves (K) based on HCCDB18 cohorts; (L) Differences in survival status of patients in different risk groups in the HCCDB18 cohort.

To further screen out prognostically relevant key genes to assess the prognosis of LIHC patients. We selected 856 genes with a correlation of greater than 0.4 with CytoTRACE in the scRNA-seq data, as well as brown module genes associated with mRNAsi. As shown in Fig 4A, a total of 16 genes related to LIHC prognosis were identified for further analysis, and they all showed prognostic significance (Fig 4B). Subsequently, the genes were narrowed down by LASSO regression analysis, and four prognostically critical genes (including STIP1, H2AFZ, BRIX1, and TUBB) were selected for the construction of prognostic models. In addition, multivariate Cox regression analysis was used to further verify the results and correlation coefficients were obtained using the formula: Risk score = (0.426 × expression of *STIP1*) + (0.203 × expression of *H2AFZ*) + (0.421 × expression of *BRIX1*) + (-0.228 × expression of *TUBB*).

The reliability of the prognostic model related to the stem cell index was evaluated by performing survival analysis. High-risk patients showed a more pronounced decrease in OS, DSS, DFEI, and PFI compared with those in the low-risk group (Fig 4E–4H). Survival difference results showed that low-risk patients accounted for a greater proportion of alive status compared to patients at high risk for LIHC (Fig 4I). Similarly, we obtained the same result in the HCCDB18 cohort (Fig 4K and 4L). In addition, we performed ROC curve analysis for the TCGA and HCCDB18 cohorts, respectively. The results showed area under curves [17] of ROC at 1, 3, and 5 years were 0.76, 0.70, and 0.69 for the TCGA cohort and 0.67, 0.73, and 0.77 for the HCCDB18 cohort, respectively (Fig 4D and 4J).

### Risk scores combined with clinical information to construct a new nomogram

**Fig 5:**
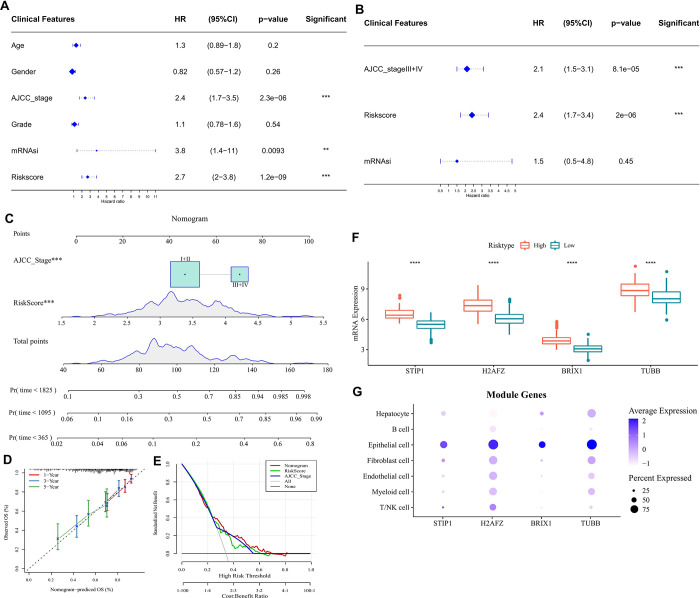
A nomogram formed based on stem cells index-related risk score signature with clinical features. Univariate (A) and multivariate (B) cox analysis of risk scores and clinical information; (C) A nomogram based on risk scores and clinical stage of AJCC; Nomogram-based calibration curves (D) and decision curves (E); (F) Differential expression of prognostic critical genes across risk groups in the TCGA cohort; (G) Expression distribution of prognostic critical genes in single-cell profile.

The association between LIHC patients and clinical information was further analyzed using univariate and multivariate Cox regression algorithms. The AJCC stage and risk score were found to be significant prognostic risk factors for patients with LIHC (Fig 5A and 5B). A nomogram was developed by combining the risk score and the AJCC stage to quantify the risk score and survival probability of LIHC patients. As shown in Fig 5C, we found that the risk score was the variable that had the largest impact on survival among all the variables. Additionally, the calibration curves indicate that the nomogram we built has good predictive performance and DCA showed the reliability of the risk model (Fig 5D and 5E). To further understand the expression of four prognostic key genes (including *STIP1*, *H2AFZ*, *BRIX1*, and *TUBB*) in the TCGA dataset and single-cell profile, respectively. As shown in Fig 5F and 5G, all four prognostic key genes showed a high expression in the LIHC high-risk group and epithelial cells.

### Differential clinical characteristics and pathways in high and low risk groups

**Fig 6:**
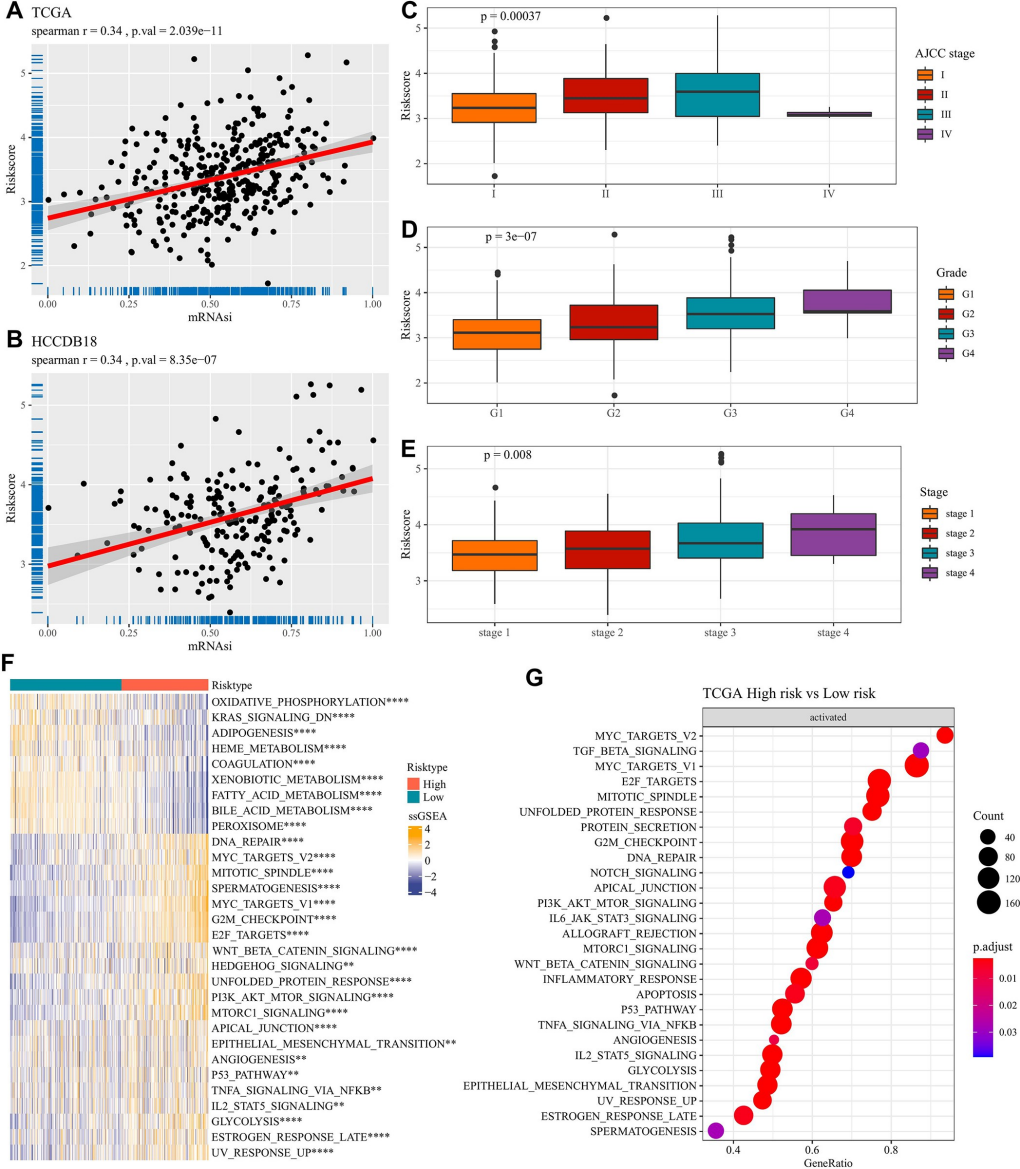
Analysis of clinical characteristics and pathway differences between LIHC risk groups. Correlation of risk scores with mRNAsi in the TCGA cohort (A) and the HCCDB18 cohort (B); Difference in risk score distribution between AJCC stage (C) and grade (D) in TCGA cohort; (E) Comparison of the distribution of risk score in HCCDB18 cohort across stages; (F) Pathway differences between risk groups in the TCGA cohort; (G) Comparison of GSEA analysis between risk groups in the TCGA cohort.

To further explore the potential impact of risk scores on LIHC patients, we first assessed the correlation between risk scores and mRNAsi in TCGA-LIHC and HCCDB18 cohort, respectively. The results showed that risk score was positively associated with the mRNAsi (Fig 6A and 6B). Next, comparison on the distribution of risk scores across stage and grade in the TCGA-LIHC and HCCDB18 cohorts demonstrated that risk scores increased with stage and grade (Fig 6C–6E). Similarly, the small number of samples in the TCGA-LIHC cohort leads to a bias in the performance in stage IV. In addition, we obtained the access information in the file "h.all.v2023.1.Hs.symbols.gmt" from the gene set enrichment analysis (GSEA) website and scored it applying the ssGSEA algorithm. There were significant differences (p<0.01) between the different risk groups of LIHC and the 30 cancer associated pathways in the TCGA cohort. As shown in Fig 6F, the LIHC low-risk group was mainly enriched in pathways related to metabolism such as bile acid metabolism and fatty acid metabolism, while the high-risk group had higher activity in cell cycle-related pathways. Interestingly, GSEA analysis showed similar results and demonstrated a significant enrichment of the LIHC high-risk group on cell cycle-related pathways (Fig 6G). Additionally, inflammatory related pathways including WNT_BETA_CATENIN_SIGNALING, TNFA_SIGNALING_VIA_NFKB, IL2_STAT5_SIGNALING were both enriched in high risk group. These findings revealed that the established risk model was closely related to cancer stemness and could well distinguish LIHC patients with different clinical information as well as disturbed pathways. Also, enriched inflammatory pathways forced us to probe the immune status in two risk groups.

#### Immune infiltration level in two risk groups

**Fig 7:**
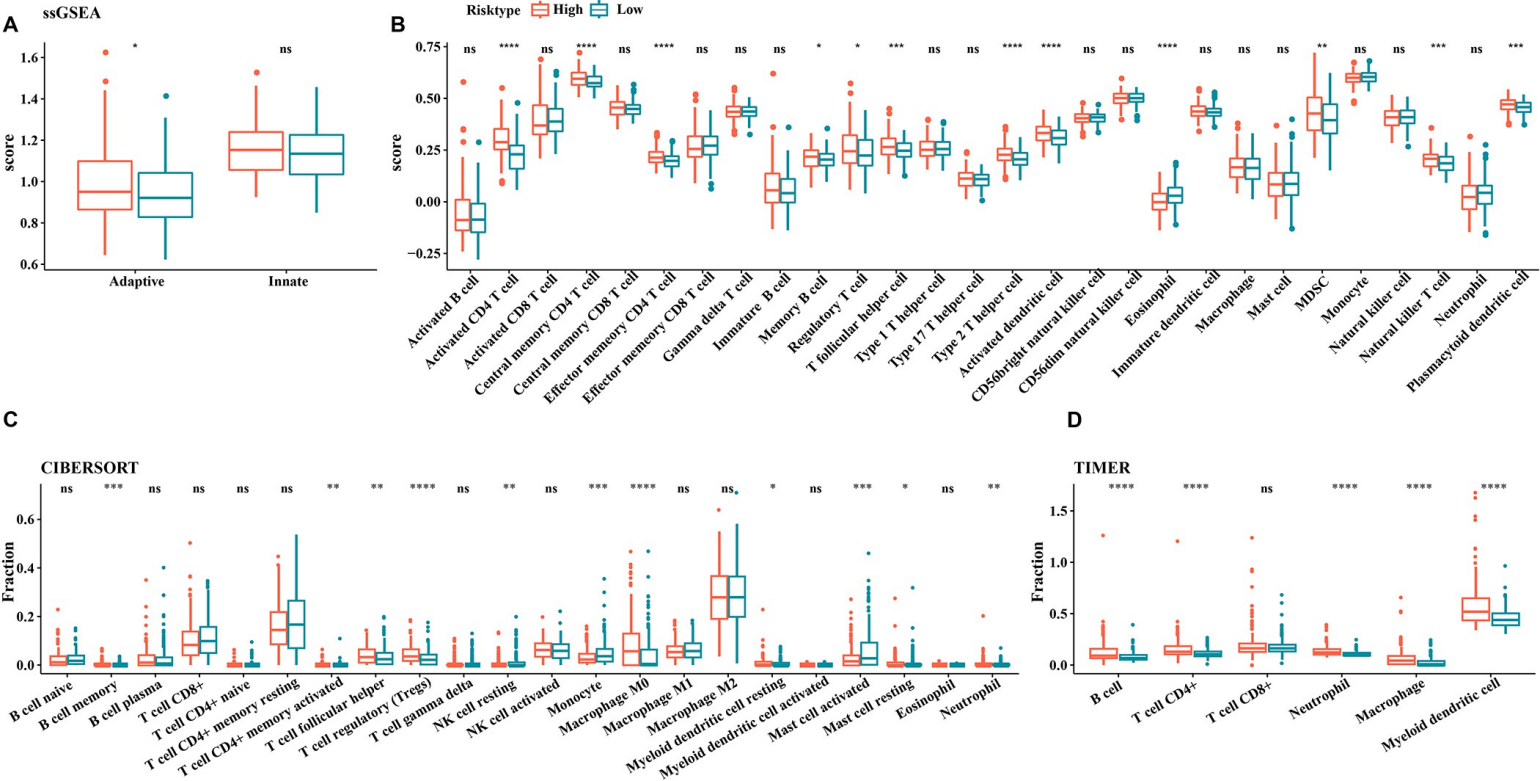
Immune infiltration analysis for two risk groups of LIHC. (A) Differences in innate and adaptive immunity between high and low risk groups; (B) The ssGSEA algorithm assesses the extent of immune cell infiltration in different risk groups; (C) CIBERSORT analysis; (D) TIMER analysis. *p < 0.05, **p < 0.01, ***p <0.001, ****p <0.0001, ns, no signicance.

To explore the differences in tumor microenvironment (TME) between patients in the high and low risk groups for LIHC, we first assessed the differences in the extent of infiltration of 28 immune cells across risk groups using the ssGSEA algorithm. Firstly, we found that patients in the high-risk group also had significantly higher adaptive immunity scores than those in the low-risk group (Fig 7A). To be more specific, the immune cell infiltration scores of patients in the high risk group of LIHC were higher than low-risk patients in most cells, such as eosinophil, natural kill cell, T follicular helper cell, activated dendritic cell, MDSC, and CD4 T cell (Fig 7B). Furthermore, relatively high proportions of immunocytes, especially macrophages were also observed in high risk groups (Fig 7C and 7D). Although these analyses were based on different algorithms, they also indicated that high risk group had higher immune infiltration levels.

### Immunotherapeutic response and drug sensitivity analysis

**Fig 8:**
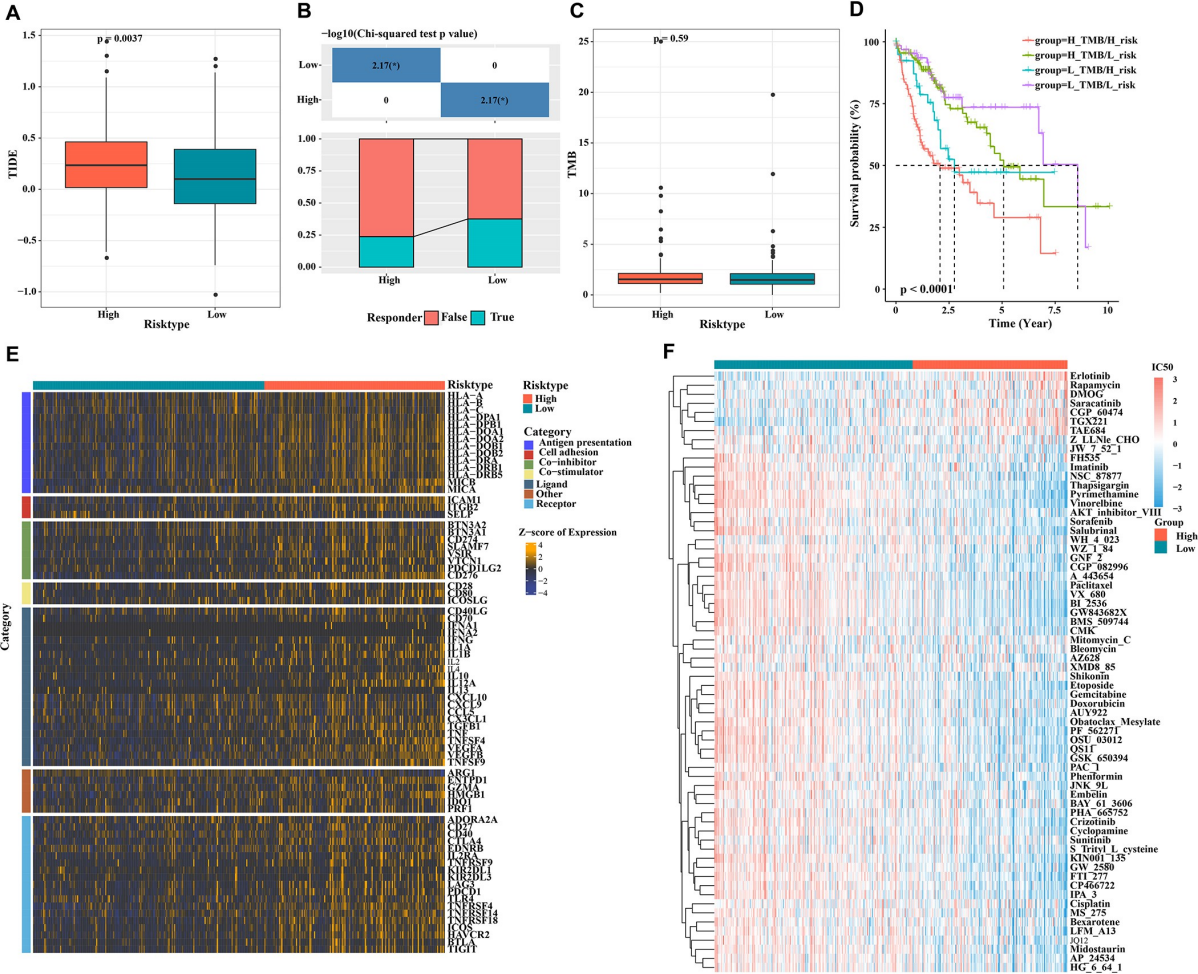
Immunotherapy and drug sensitivity in different risk groups of LIHC. (A) TIDE algorithm assesses immune escape potential in different risk groups; (B) Analysis of differences in immune response between high and low risk groups; (C) There was no significant difference in TMB levels between the high and low risk groups; (D) KM curves in the high- and low-risk groups after combination with TMB; (E) Expression levels of immunomodulator-related genes in different risk groups; (F) Analysis of differences in drug sensitivity.

Notably, LIHC low-risk patients had significantly lower TIDE scores than patients in the high-risk group (Fig 8A), and the percentage of immune responses was higher in low-risk patients than in the high-risk group (Fig 8B). This suggests that patients in the low-risk LIHC group were more likely to benefit from immunotherapy. TMB is also considered as a biomarker of tumor immunotherapy response rate and patients with high TMB benefiting more from immunotherapy as well as longer OS [33]. Nonetheless, as shown in Fig 8C, TMB did not differ significantly between LIHC patients in different risk groups (p = 0.59). However, we found that in KM survival analysis, when combining with risk score, low risk and low TMB patients displayed the longest OS, while high risk and high TMB patients exhibited the shortest OS (Fig 8D). Integrating the results of TIDE analysis, the findings in Fig 7F indicated that the established risk model seemed more effective for immunotherapy evaluation of LIHC patients. The expression of immunomodulator-associated genes in patients in different risk groups of LIHC also showed significantly high expression in the high-risk group (Fig 8E). In addition, we assessed the sensitivity of different risk groups to 33 common chemotherapeutic drugs using the "pRRophetic" package in R. The results showed that patients in the LIHC high-risk group were sensitive to 29 drugs, such as Sunitinib, Etoposide, Gemcitabine, Doxorubicin, Thapsigargin, Pyrimethamine, and Vinorelbine. And the lower IC50 values for Erlotinib, TGX221, CGP60474, and Saracatinib in the low-risk group were even lower, meaning that patients with lower risk scores may benefit more from these drugs (Fig 8F). Overall, these results suggest that CSCs characteristics can predict treatment sensitivity in patients with LIHC.

## Discussion

LIHC is a combined disease with high mortality and morbidity [34]. Notably, CSCs have been demonstrated to be connected with the return, spread, and resistance to local and systemic therapy in hepatocellular carcinoma [35]. CSCs have the ability to generate an immunosuppressive microenvironment through both inherent and extrinsic mechanisms, which consequently triggers ineffective anti-tumor immune responses [36]. Despite the use of certain immunotherapies, like monoclonal antibodies for programmed cell death ligand 1 (PD-L1) and programmed cell death protein 1 (PD-1), in a wide array of solid tumors (including LIHC), a majority of patients still display progressed and metastasized tumors following treatment [37,38]. Given that CSCs serve as reservoirs for hepatocellular carcinoma progression and metastasis, targeted therapy utilizing tumor stem cells might offer a new approach against LIHC.

The prediction of cell differentiation status can now be achieved through the use of CytoTRACE, a novel computational framework developed by Gulati et al [12]. In a new study, Bian et al. investigated the interaction mechanism between the immunogenicity of CSCs and their stemness in intrahepatic cholangiocarcinoma using CytoTRACE. The outcomes of their research highlighted that CSCs with heightened stemness possess inherent immune features, enabling them to evade immune surveillance [13]. In addition, the ability to measure cancer stemness is exhibited by OCLR, an algorithm that employs stem cell samples from the PCBC dataset for training the mRNA expression-based stemness signature [14,39]. Numerous investigations have been carried out to investigate the clinical and biological importance of transcriptome stemness features. These studies have involved the integration of various datasets and have aimed to confirm the applicability of mRNAsi across different tumor types [19,40,41]. Specifically, in LIHC, Bai et al. demonstrated that mRNAsi is overexpressed in hepatocellular carcinoma tissues and identified three important modules and 21 key genes by WGCNA. They suggested that these key genes identified through the mRNA expression network may be potential therapeutic targets for inhibiting cancer cell stemness in LIHC [42].

Unlike previous studies, the present study was conducted by combining CytoTRACE score and mRNAsi to identify biomarkers of LIHC prognosis more efficiently and thus improve the precision treatment of LIHC in the future. In this regard, we identified four key prognostic genes (*STIP1*, *H2AFZ*, *BRIX1*, and *TUBB*) by selecting the brown module genes with a correlation to CytoTRACE score greater than 0.4 and mostly relevant to mRNAsi. *STIP1* is thought to be involved in mediating the process of tumor proliferation, migration, and invasion [43–45], and it is capable of being secreted by a wide range of cancer cells (including hepatocellular carcinoma cells) and can act as a cytokine in order to regulate tumor progression [46–48]. Ma et al. indicated that detection of *STIP1* in serum may be helpful in early clinical decision-making in HCC. Because they identified *STIP1* as an independent predictor of HCC after treatment with rancatheter arterial chemoembolization by KM, log-rank and Cox regression analysis [49]. Furthermore, it was observed that higher *STIP1* expression was significantly correlated with a shortened period of recurrence-free survival [50]. Our work further supported the significance of *STIP1* in LIHC diagnosis as a promising marker. *H2AFZ* is overexpressed in a variety of tumors, including bladder cancer [51], breast cancer [52], and prostate cancer [53]. Dong et al. showed that overexpression of *H2AFZ* is associated with poor prognosis and tumor malignancy in HCC patients and that its overexpression is regulated by TP53 mutations, which leads to the development of a rapidly proliferative phenotype in HCC cells [54]. Additionally, it has been reported that *H2AFZ* is able to accelerate cell cycle transition during hepatocarcinogenesis and plays a key role in epithelial-mesenchymal transition (EMT), which in turn has oncogenic potential [55]. Notably, in our study, we observed that both the set of genes most strongly associated with mRNAsi and the patients in the LIHC high-risk group had pathway enrichment results suggesting cell cycle relevance. This suggests that these results may be due to the presence of *H2AFZ*. Ge et al. concluded that *BRIX1* mRNA levels are higher in colorectal cancer (CRC) tissues than in normal tissues, and that the expression of *BRIX1* correlates with CRC tumorstage, which is a risk factor for overall survival in patients with CRC [56]. Wang and his colleagues screened several differentially expressed genes (including *BRIX1*) and they proposed that these genes may contribute to the clinical treatment of HCC as potential therapeutic targets [57]. *TUBB* belongs to the β-tubulin family, which contains 10 isoforms [58]. *TUBB* is involved in the cancer cell cycle in a variety of cancers, and its mRNA levels is correlated with an unfavorable cancer prognosis [59–61]. However, reports of *TUBB* in LIHC are lacking. This requires more in-depth studies on the mechanism of action of *TUBB* in LIHC in the future. The above results indicated that the risk model based on these 4 genes had a strong reliability for LIHC patient’s diagnosis and prognosis, and their detection in biological samples such as serum [62] or urine may be prospective.

Differences in TME between risk groups allow us to see the potential value of the stemness index in predicting immunotherapy response. In the TME, immune cells usually have the capability to promptly identify and eliminate tumor cells. However, tumor cells possess the ability to generate immune suppression within the TME, resulting in their evasion from the immune response through a multifaceted regulatory network [63,64]. Immunosuppressive TME consists of a number of immunosuppressive cells, such as regulatory T cells, myeloid-derived suppressor cells (MDSCs), tumor-associated macrophages [65]. In our study, regulatory T cells and MDSCs were highly expressed in high-risk patients compared to the low-risk group, indicating greater immunosuppression in the high-risk group. Additionally, the TIDE score, immune response and TMB suggest that low-risk patients with LIHC may do better with immunotherapy. Specifically, LIHC high-risk patients may benefit more from chemotherapeutic agents.

Nevertheless, our research also has constraints. First, the specific mechanisms of key genes associated with stemness index in LIHC still need to be validated by further in vivo and in vitro experiments. Secondly, it is imperative to incorporate more comprehensive clinical case traits in this analysis to enable a more thorough assessment of the prognostic model’s clinical significance.

## Conclusion

To summarize, we have discovered four genes linked to CSCs by integrating two innovative techniques to evaluate the stemness of cells. Additionally, we have formulated a model utilizing these identified genes, which holds promise as a reliable prognostic indicator for patients diagnosed with LIHC. The generated risk score exhibits the potential to function independently as a prognosis predictor for LIHC patients. This score can offer valuable guidance to patients seeking optimal benefits from diverse immunotherapies.

## Supporting information

S1 Fig Construction of mRNAsi related module genes using WGCNA. (A) Analysis of scale-free exponent and average connectivity of various soft threshold powers; (B) Cluster dendrogram of the co-expression network modules; (C) Module thresholds for WGCNA analysis; (D) The number of genes in each module.
(PDF)

S2 Fig The number of genes in each module using WGCNA. (PDF)

S1 Table Detailed genes in brown module using WGCNA. (XLSX)

## Abbreviations

LIHC: Liver hepatocellular carcinoma
CSCs: cancer stem cell-associated genes
scRNA-seq: single-cell RNA sequencing
mRNAsi: mRNA expression-based stemness index
WGCNA: weighted co-expression network analysis
TCGA: The Cancer Genome AtlasAUC, area under ROC curve
CDF: cumulative distribution functio
DCA: Decision curve analysis
GEO: Gene Expression Omnibus
HR: hazard ratio
PCA: principal component analysis
UMAP: universal manifold approximation and projection
LASSO: least absolute shrinkage and selection operator
OS: overall survival
GO: Gene Ontology
KEGG: Kyoto Encyclopedia of Genes and Genomes
OCLR: One-Class Logistic Regression
PCBC: Progenitor Cell Biology Consortium
ROC: receiver operating characteristic analysis
KM: Kaplan-Meier
DSS: disease-specific survival
DFI: disease-free interval
PFI: progression-free interval
PVTT: portal vein tumor thrombosis
ssGSEA: single-sample gene set enrichment analysis
TIDE: Tumor Immune Dysfunction and Exclusion
TME: tumor microenvironment
TMB: tumor mutation burden
STIP1: stress-induced phosphoprotein 1
TUBB: Tubulin beta class I gene

## References

[1] Sung H, Ferlay J, Siegel RL, Laversanne M, Soerjomataram I, Jemal A, et al. Global Cancer Statistics 2020: GLOBOCAN Estimates of Incidence and Mortality Worldwide for 36 Cancers in 185 Countries. CA Cancer J Clin. 2021;71(3):209–49. doi: 10.3322/caac.21660 33538338

[2] Forner A, Reig M, Bruix J. Hepatocellular carcinoma. Lancet. 2018;391(10127):1301–14. doi: 10.1016/S0140-6736(18)30010-2 29307467

[3] Prieto J, Melero I, Sangro B. Immunological landscape and immunotherapy of hepatocellular carcinoma. Nat Rev Gastroenterol Hepatol. 2015;12(12):681–700. doi: 10.1038/nrgastro.2015.173 26484443

[4] Shu X, Wang Q, Wu Q. The Eph/Ephrin System in Hepatocellular Carcinoma: Functional Roles and Potential Therapeutic Targets. Oncologie. 2022;24(3):427–39.

[5] Behrens S, Wang XW. Dissecting intratumor heterogeneity in HCC: new research strategies and clinical implications. Carcinogenesis. 2022;43(12):1103–9. doi: 10.1093/carcin/bgac099 36512331 PMC10122425

[6] Clevers H. The cancer stem cell: premises, promises and challenges. Nature Medicine. 2011;17(3):313–9. doi: 10.1038/nm.2304 21386835

[7] Friedmann-Morvinski D, Verma IM. Dedifferentiation and reprogramming: origins of cancer stem cells. EMBO Rep. 2014;15(3):244–53. doi: 10.1002/embr.201338254 24531722 PMC3989690

[8] Aghaalikhani N, Rashtchizadeh N, Shadpour P, Allameh A, Mahmoodi M. Cancer stem cells as a therapeutic target in bladder cancer. J Cell Physiol. 2019;234(4):3197–206. doi: 10.1002/jcp.26916 30471107

[9] Schulte LA, López-Gil JC, Sainz B Jr., Hermann PC. The Cancer Stem Cell in Hepatocellular Carcinoma. Cancers (Basel). 2020;12(3). doi: 10.3390/cancers12030684 32183251 PMC7140091

[10] Blanpain C, Mohrin M, Sotiropoulou PA, Passegué E. DNA-damage response in tissue-specific and cancer stem cells. Cell Stem Cell. 2011;8(1):16–29. doi: 10.1016/j.stem.2010.12.012 21211780

[11] Zheng H, Pomyen Y, Hernandez MO, Li C, Livak F, Tang W, et al. Single-cell analysis reveals cancer stem cell heterogeneity in hepatocellular carcinoma. Hepatology. 2018;68(1):127–40. doi: 10.1002/hep.29778 29315726 PMC6033650

[12] Gulati GS, Sikandar SS, Wesche DJ, Manjunath A, Bharadwaj A, Berger MJ, et al. Single-cell transcriptional diversity is a hallmark of developmental potential. Science. 2020;367(6476):405–11. doi: 10.1126/science.aax0249 31974247 PMC7694873

[13] Bian J, Fu J, Wang X, Lee J, Brar G, Escorcia FE, et al. Characterization of Immunogenicity of Malignant Cells with Stemness in Intrahepatic Cholangiocarcinoma by Single-Cell RNA Sequencing. Stem Cells International. 2022;2022:1–14. doi: 10.1155/2022/3558200 35530414 PMC9076354

[14] Malta TM, Sokolov A, Gentles AJ, Burzykowski T, Poisson L, Weinstein JN, et al. Machine Learning Identifies Stemness Features Associated with Oncogenic Dedifferentiation. Cell. 2018;173(2):338–54.e15. doi: 10.1016/j.cell.2018.03.034 29625051 PMC5902191

[15] Lu Y, Yang A, Quan C, Pan Y, Zhang H, Li Y, et al. A single-cell atlas of the multicellular ecosystem of primary and metastatic hepatocellular carcinoma. Nat Commun. 2022;13(1):4594. doi: 10.1038/s41467-022-32283-3 35933472 PMC9357016

[16] Yan D, Li C, Zhou Y, Yan X, Zhi W, Qian H, et al. Exploration of Combinational Therapeutic Strategies for HCC Based on TCGA HCC Database. Oncologie. 2022;24(1):101–11.

[17] Stuart T, Butler A, Hoffman P, Hafemeister C, Papalexi E, Mauck WM 3rd, et al. Comprehensive Integration of Single-Cell Data. Cell. 2019;177(7):1888–902.e21. doi: 10.1016/j.cell.2019.05.031 31178118 PMC6687398

[18] Chen S, Zhu G, Yang Y, Wang F, Xiao YT, Zhang N, et al. Single-cell analysis reveals transcriptomic remodellings in distinct cell types that contribute to human prostate cancer progression. Nat Cell Biol. 2021;23(1):87–98. doi: 10.1038/s41556-020-00613-6 33420488

[19] Wang Z, Wang Y, Yang T, Xing H, Wang Y, Gao L, et al. Machine learning revealed stemness features and a novel stemness-based classification with appealing implications in discriminating the prognosis, immunotherapy and temozolomide responses of 906 glioblastoma patients. Brief Bioinform. 2021;22(5). doi: 10.1093/bib/bbab032 33839757 PMC8425448

[20] Fang Y, Huang S, Han L, Wang S, Xiong B. Comprehensive Analysis of Peritoneal Metastasis Sequencing Data to Identify LINC00924 as a Prognostic Biomarker in Gastric Cancer. Cancer Manag Res. 2021;13:5599–611. doi: 10.2147/CMAR.S318704 34285580 PMC8285530

[21] Langfelder P, Horvath S. WGCNA: an R package for weighted correlation network analysis. BMC Bioinformatics. 2008;9:559. doi: 10.1186/1471-2105-9-559 19114008 PMC2631488

[22] Yu G, Wang LG, Han Y, He QY. clusterProfiler: an R package for comparing biological themes among gene clusters. Omics. 2012;16(5):284–7. doi: 10.1089/omi.2011.0118 22455463 PMC3339379

[23] Kanehisa M, Goto S. KEGG: Kyoto Encyclopedia of Genes and Genomes. Nucleic Acids Research. 2000;28(1):27–30. doi: 10.1093/nar/28.1.27 10592173 PMC102409

[24] Simon N, Friedman J, Hastie T, Tibshirani R. Regularization Paths for Cox’s Proportional Hazards Model via Coordinate Descent. Journal of Statistical Software. 2011;39(5). doi: 10.18637/jss.v039.i05 27065756 PMC4824408

[25] He S, Jiang R, Sun H, Yang J, Ye C, Liu W, et al. Surgical efficacy and survival prediction of patients with unspecified malignant bone tumors. BMC Cancer. 2022;22(1):1078. doi: 10.1186/s12885-022-10153-x 36266614 PMC9583561

[26] Charoentong P, Finotello F, Angelova M, Mayer C, Efremova M, Rieder D, et al. Pan-cancer Immunogenomic Analyses Reveal Genotype-Immunophenotype Relationships and Predictors of Response to Checkpoint Blockade. Cell Rep. 2017;18(1):248–62. doi: 10.1016/j.celrep.2016.12.019 28052254

[27] He Y, Jiang Z, Chen C, Wang X. Classification of triple-negative breast cancers based on Immunogenomic profiling. J Exp Clin Cancer Res. 2018;37(1):327. doi: 10.1186/s13046-018-1002-1 30594216 PMC6310928

[28] Chen B, Khodadoust MS, Liu CL, Newman AM, Alizadeh AA. Profiling Tumor Infiltrating Immune Cells with CIBERSORT. Methods Mol Biol. 2018;1711:243–59. doi: 10.1007/978-1-4939-7493-1_12 29344893 PMC5895181

[29] Li T, Fu J, Zeng Z, Cohen D, Li J, Chen Q, et al. TIMER2.0 for analysis of tumor-infiltrating immune cells. Nucleic Acids Res. 2020;48(W1):W509–w14. doi: 10.1093/nar/gkaa407 32442275 PMC7319575

[30] Jiang P, Gu S, Pan D, Fu J, Sahu A, Hu X, et al. Signatures of T cell dysfunction and exclusion predict cancer immunotherapy response. Nat Med. 2018;24(10):1550–8. doi: 10.1038/s41591-018-0136-1 30127393 PMC6487502

[31] Geeleher P, Cox N, Huang RS. pRRophetic: an R package for prediction of clinical chemotherapeutic response from tumor gene expression levels. PLoS One. 2014;9(9):e107468. doi: 10.1371/journal.pone.0107468 25229481 PMC4167990

[32] Thorsson V, Gibbs DL, Brown SD, Wolf D, Bortone DS, Ou Yang TH, et al. The Immune Landscape of Cancer. Immunity. 2018;48(4):812–30.e14. doi: 10.1016/j.immuni.2018.03.023 29628290 PMC5982584

[33] Rizzo A, Ricci AD, Brandi G. PD-L1, TMB, MSI, and Other Predictors of Response to Immune Checkpoint Inhibitors in Biliary Tract Cancer. Cancers (Basel). 2021;13(3). doi: 10.3390/cancers13030558 33535621 PMC7867133

[34] Ryerson AB, Eheman CR, Altekruse SF, Ward JW, Jemal A, Sherman RL, et al. Annual Report to the Nation on the Status of Cancer, 1975–2012, featuring the increasing incidence of liver cancer. Cancer. 2016;122(9):1312–37. doi: 10.1002/cncr.29936 26959385 PMC4840031

[35] Lee TK, Guan XY, Ma S. Cancer stem cells in hepatocellular carcinoma—from origin to clinical implications. Nat Rev Gastroenterol Hepatol. 2022;19(1):26–44. doi: 10.1038/s41575-021-00508-3 34504325

[36] Ruiu R, Tarone L, Rolih V, Barutello G, Bolli E, Riccardo F, et al. Cancer stem cell immunology and immunotherapy: Harnessing the immune system against cancer’s source. Prog Mol Biol Transl Sci. 2019;164:119–88. doi: 10.1016/bs.pmbts.2019.03.008 31383404

[37] Greten TF, Lai CW, Li G, Staveley-O’Carroll KF. Targeted and Immune-Based Therapies for Hepatocellular Carcinoma. Gastroenterology. 2019;156(2):510–24. doi: 10.1053/j.gastro.2018.09.051 30287171 PMC6340758

[38] Ribas A, Wolchok JD. Cancer immunotherapy using checkpoint blockade. Science. 2018;359(6382):1350–5. doi: 10.1126/science.aar4060 29567705 PMC7391259

[39] Sokolov A, Paull EO, Stuart JM. ONE-CLASS DETECTION OF CELL STATES IN TUMOR SUBTYPES. Pac Symp Biocomput. 2016;21:405–16. 26776204 PMC4856035

[40] Stahl D, Knoll R, Gentles AJ, Vokuhl C, Buness A, Gütgemann I. Prognostic Gene Expression, Stemness and Immune Microenvironment in Pediatric Tumors. Cancers (Basel). 2021;13(4). doi: 10.3390/cancers13040854 33670534 PMC7922568

[41] Zhang C, Chen T, Li Z, Liu A, Xu Y, Gao Y, et al. Depiction of tumor stemlike features and underlying relationships with hazard immune infiltrations based on large prostate cancer cohorts. Brief Bioinform. 2021;22(3). doi: 10.1093/bib/bbaa211 32856039

[42] Bai KH, He SY, Shu LL, Wang WD, Lin SY, Zhang QY, et al. Identification of cancer stem cell characteristics in liver hepatocellular carcinoma by WGCNA analysis of transcriptome stemness index. Cancer Medicine. 2020;9(12):4290–8. doi: 10.1002/cam4.3047 32311840 PMC7300398

[43] Van Simaeys D, Turek D, Champanhac C, Vaizer J, Sefah K, Zhen J, et al. Identification of cell membrane protein stress-induced phosphoprotein 1 as a potential ovarian cancer biomarker using aptamers selected by cell systematic evolution of ligands by exponential enrichment. Anal Chem. 2014;86(9):4521–7. doi: 10.1021/ac500466x 24654750 PMC4018121

[44] Tsai CL, Tsai CN, Lin CY, Chen HW, Lee YS, Chao A, et al. Secreted stress-induced phosphoprotein 1 activates the ALK2-SMAD signaling pathways and promotes cell proliferation of ovarian cancer cells. Cell Rep. 2012;2(2):283–93. doi: 10.1016/j.celrep.2012.07.002 22884369

[45] Huang L, Zhai E, Cai S, Lin Y, Liao J, Jin H, et al. Stress-inducible Protein-1 promotes metastasis of gastric cancer via Wnt/β-catenin signaling pathway. J Exp Clin Cancer Res. 2018;37(1):6. 29335007 10.1186/s13046-018-0676-8 PMC5769340

[46] Krafft U, Tschirdewahn S, Hess J, Harke NN, Hadaschik BA, Nyirády P, et al. STIP1 Tissue Expression Is Associated with Survival in Chemotherapy-Treated Bladder Cancer Patients. Pathol Oncol Res. 2020;26(2):1243–9. doi: 10.1007/s12253-019-00689-y 31250373

[47] Wang HS, Tsai CL, Chang PY, Chao A, Wu RC, Chen SH, et al. Positive associations between upregulated levels of stress-induced phosphoprotein 1 and matrix metalloproteinase-9 in endometriosis/adenomyosis. PLoS One. 2018;13(1):e0190573. doi: 10.1371/journal.pone.0190573 29304094 PMC5755831

[48] Chen Z, Xu L, Su T, Ke Z, Peng Z, Zhang N, et al. Autocrine STIP1 signaling promotes tumor growth and is associated with disease outcome in hepatocellular carcinoma. Biochem Biophys Res Commun. 2017;493(1):365–72. doi: 10.1016/j.bbrc.2017.09.016 28887036

[49] Ma XL, Tang WG, Yang MJ, Xie SH, Wu ML, Lin G, et al. Serum STIP1, a Novel Indicator for Microvascular Invasion, Predicts Outcomes and Treatment Response in Hepatocellular Carcinoma. Front Oncol. 2020;10:511. doi: 10.3389/fonc.2020.00511 32426271 PMC7212360

[50] Su T, Liao J, Dai Z, Xu L, Chen S, Wang Y, et al. Stress-induced phosphoprotein 1 mediates hepatocellular carcinoma metastasis after insufficient radiofrequency ablation. Oncogene. 2018;37(26):3514–27. doi: 10.1038/s41388-018-0169-4 29559743

[51] Kim K, Punj V, Choi J, Heo K, Kim JM, Laird PW, et al. Gene dysregulation by histone variant H2A.Z in bladder cancer. Epigenetics Chromatin. 2013;6(1):34. doi: 10.1186/1756-8935-6-34 24279307 PMC3853418

[52] Sawant A, Hensel JA, Chanda D, Harris BA, Siegal GP, Maheshwari A, et al. Depletion of plasmacytoid dendritic cells inhibits tumor growth and prevents bone metastasis of breast cancer cells. J Immunol. 2012;189(9):4258–65. doi: 10.4049/jimmunol.1101855 23018462 PMC3531993

[53] Tyagi M, Cheema MS, Dryhurst D, Eskiw CH, Ausió J. Metformin alters H2A.Z dynamics and regulates androgen dependent prostate cancer progression. Oncotarget. 2018;9(97):37054–68. doi: 10.18632/oncotarget.26457 30651935 PMC6319340

[54] Dong M, Chen J, Deng Y, Zhang D, Dong L, Sun D. H2AFZ Is a Prognostic Biomarker Correlated to TP53 Mutation and Immune Infiltration in Hepatocellular Carcinoma. Front Oncol. 2021;11:701736. doi: 10.3389/fonc.2021.701736 34760688 PMC8573175

[55] Yang HD, Kim PJ, Eun JW, Shen Q, Kim HS, Shin WC, et al. Oncogenic potential of histone-variant H2A.Z.1 and its regulatory role in cell cycle and epithelial-mesenchymal transition in liver cancer. Oncotarget. 2016;7(10):11412–23. doi: 10.18632/oncotarget.7194 26863632 PMC4905482

[56] Ge J, Huang X, Wang P, Lu C. Expression of biogenesis of ribosomes BRX1 is associated with malignant progression and prognosis in colorectal cancer. Transl Cancer Res. 2020;9(9):5595–602. doi: 10.21037/tcr-20-2564 35117923 PMC8798812

[57] Wang L, Zhang Z, Li Y, Wan Y, Xing B. Integrated bioinformatic analysis of RNA binding proteins in hepatocellular carcinoma. Aging (Albany NY). 2020;13(2):2480–505. doi: 10.18632/aging.202281 33411682 PMC7880356

[58] Findeisen P, Mühlhausen S, Dempewolf S, Hertzog J, Zietlow A, Carlomagno T, et al. Six subgroups and extensive recent duplications characterize the evolution of the eukaryotic tubulin protein family. Genome Biol Evol. 2014;6(9):2274–88. doi: 10.1093/gbe/evu187 25169981 PMC4202323

[59] Bakker EY, Fujii M, Krstic-Demonacos M, Demonacos C, Alhammad R. Protein disulfide isomerase A1‑associated pathways in the development of stratified breast cancer therapies. Int J Oncol. 2022;60(2). 10.3892/ijo.2022.5306 PMC8776328 35014681

[60] Ma Y, Dai H, Kong X, Wang L. Impact of thawing on reference gene expression stability in renal cell carcinoma samples. Diagn Mol Pathol. 2012;21(3):157–63. doi: 10.1097/PDM.0b013e31824d3435 22847160

[61] Yang J, Yang J, Gao Y, Zhao L, Liu L, Qin Y, et al. Identification of potential serum proteomic biomarkers for clear cell renal cell carcinoma. PLoS One. 2014;9(11):e111364. doi: 10.1371/journal.pone.0111364 25368985 PMC4219714

[62] Wang X, Xu J, Gu Q, Tang D, Ji H, Ju S, et al. A UHPLC/MS/MS Assay Based on an Isotope-Labeled Peptide for Sensitive miR-21 Detection in HCC Serum. Oncologie. 2022;24(3):513–26.

[63] Joyce JA, Fearon DT. T cell exclusion, immune privilege, and the tumor microenvironment. Science. 2015;348(6230):74–80. doi: 10.1126/science.aaa6204 25838376

[64] Jiang X, Wang J, Deng X, Xiong F, Ge J, Xiang B, et al. Role of the tumor microenvironment in PD-L1/PD-1-mediated tumor immune escape. Mol Cancer. 2019;18(1):10. doi: 10.1186/s12943-018-0928-4 30646912 PMC6332843

[65] Zeng C, He R, Dai Y, Lu X, Deng L, Zhu Q, et al. Identification of TGF-β signaling-related molecular patterns, construction of a prognostic model, and prediction of immunotherapy response in gastric cancer. Frontiers in Pharmacology. 2022;13. 10.3389/fphar.2022.1069204 PMC9715605 36467074

