# Supplementary material for: Integration of single-cell RNA-seq and bulk RNA-seq to construct liver hepatocellular carcinoma stem cell signatures to explore their impact on patient prognosis and treatment
Source: PLoS One. 2024 Apr 18;19(4):e0298004. doi: 10.1371/journal.pone.0298004 (PMC11025768; doi:10.1371/journal.pone.0298004)
Supplement: S1 Fig — (A) Analysis of scale-free exponent and average connectivity of various soft threshold powers; (B) Cluster dendrogram of the co-expression network modules; (C) Module thresholds for WGCNA analysis; (D) The number of genes in each module. (PDF) [file pone.0298004.s001.pdf]

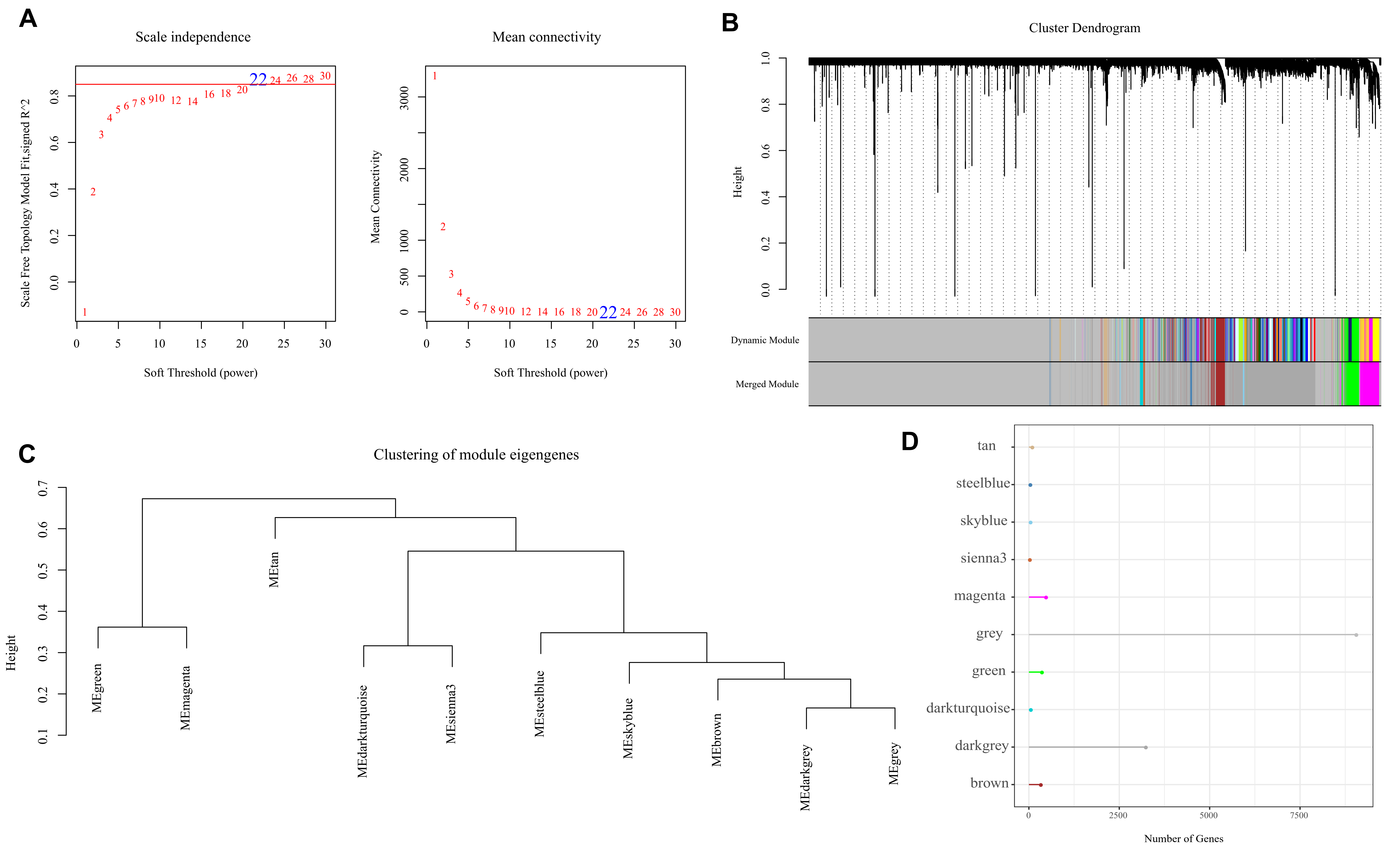
A
Scale independence
Scale Free Topology Model Fit,signed R^2
Soft Threshold (power)
Mean connectivity
Mean Connectivity
Soft Threshold (power)
B
Cluster Dendrogram
Height
Dynamic Module
Merged Module
C
Clustering of module eigengenes
Height
MEgreen
MEmagenta
MEtan
MEdarkturquoise
MEsienna3
MEsteelblue
MEskyblue
MEbrown
MEdarkgrey
MEgrey
D
tan
steelblue
skyblue
sienna3
magenta
grey
green
darkturquoise
darkgrey
brown
Number of Genes
