# Supplementary figures and images for: Integration of single-cell RNA-seq and bulk RNA-seq to construct liver hepatocellular carcinoma stem cell signatures to explore their impact on patient prognosis and treatment
Source: PLoS One. 2024 Apr 18;19(4):e0298004. doi: 10.1371/journal.pone.0298004 (PMC11025768; doi:10.1371/journal.pone.0298004)

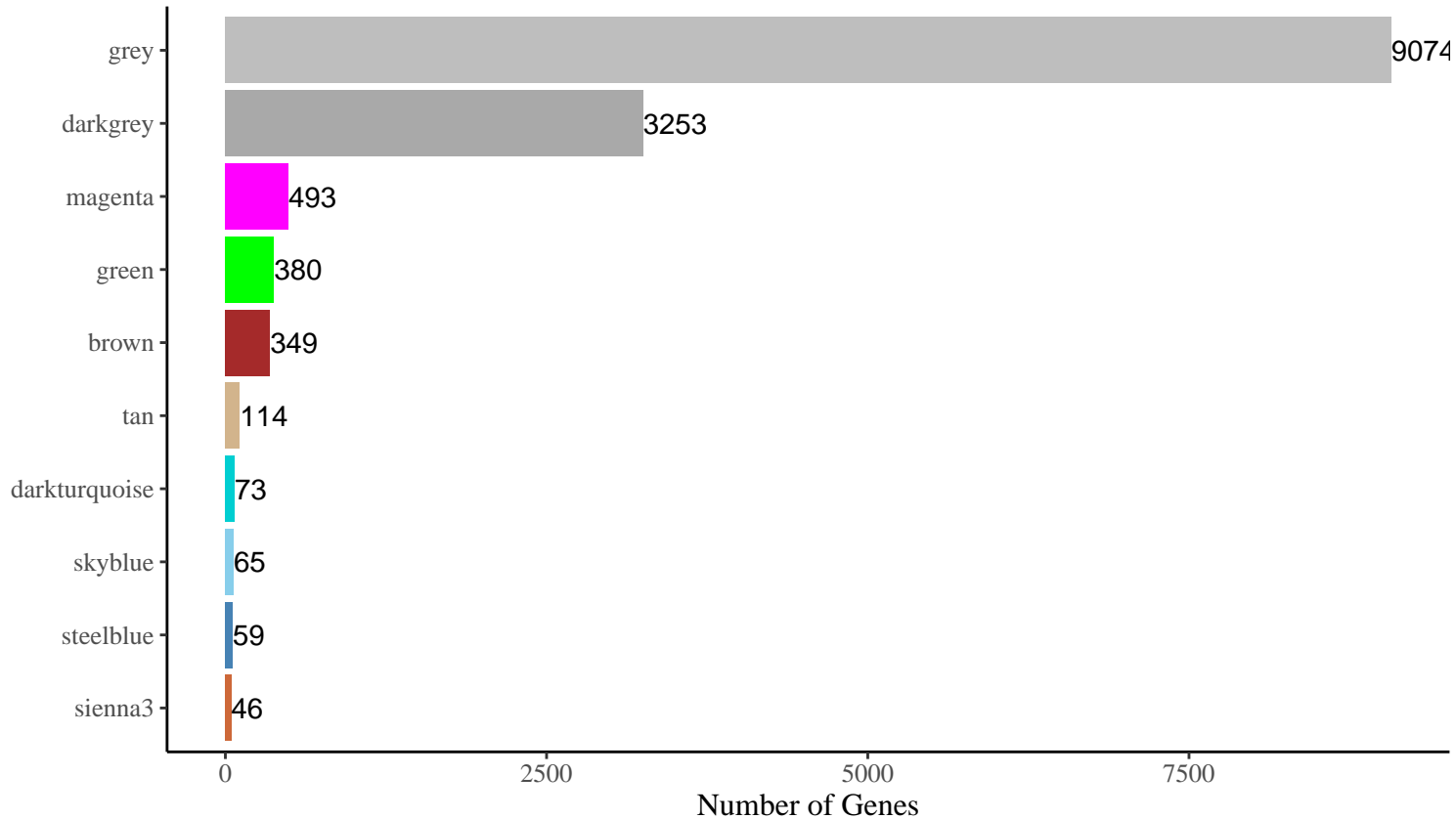

Supplement: S2 Fig — (PDF) [file pone.0298004.s002.pdf]
